# Long working hours and the risk of hypothyroidism in healthy Korean workers: a cohort study

**DOI:** 10.4178/epih.e2022104

**Published:** 2022-11-08

**Authors:** Yesung Lee, Woncheol Lee, Hyoung-Ryoul Kim

**Affiliations:** 1 Department of Occupational and Environmental Medicine, Kangbuk Samsung Hospital, Sungkyunkwan University School of Medicine, Seoul, Korea; 2 Department of Occupational and Environmental Medicine, College of Medicine, The Catholic University of Korea, Seoul, Korea

**Keywords:** Hypothyroidism, Long working hours, Overwork, Thyroid, Longitudinal studies, Cohort studies

## Abstract

**OBJECTIVES:**

Long working hours have been reported to cause various health problems, but are currently practiced in many countries. Building upon a previous cross-sectional study, the authors aimed to elucidate the causal relationship between long working hours and hypothyroidism through a longitudinal study.

**METHODS:**

Data were collected at baseline from 45,259 participants without thyroid disease and with consistent weekly working hours (36–40, 41–52, 53–60, and >60 hours) during the follow-up period. Hypothyroidism was defined using the reference limits of serum thyroid-stimulating hormone and free thyroxine levels. By estimating hazard ratios (HRs) and 95% confidence intervals (CIs) using Cox proportional hazards regression analysis, the risk of incident hypothyroidism was evaluated with 36–40 hours of work per week as the reference.

**RESULTS:**

During 138,261.7 person-years of follow-up, 2,914 participants developed hypothyroidism (incidence density, 2.11/10^2^ person-years). The multivariable-adjusted HRs of incident hypothyroidism for 41–52 hours, 53–60 hours, and >60 hours of work per week were 1.13 (95% CI, 1.03 to 1.24), 2.53 (95% CI, 2.17 to 2.95), and 2.57 (95% CI, 2.09 to 3.15), respectively. In dose-response analyses, long working hours had an approximately linear relationship with hypothyroidism incidence. The risk of incident hypothyroidism in those who worked 53–60 hours and >60 hours per week compared with the reference group was significantly higher among the older age group (≥36 years, stratified by median age), men, and daytime workers.

**CONCLUSIONS:**

This large-scale cohort study demonstrated the association between long working hours and an increased risk of incident hypothyroidism with a dose-response relationship.

## INTRODUCTION

Occupational factors, such as long working hours, have been recognized as significant issues adversely affecting workers’ health [1–3]. In addition to threatening the health of workers, long working hours also reduce work efficiency, which can have a negative impact on an entire industry [4]. Nevertheless, there are many countries where long working hours are practiced. According to a report from the Organization for Economic Cooperation and Development (OECD) in 2020, the average annual hours actually worked was almost 2,000 hours in Colombia, Mexico, Costa Rica, and Korea, whereas the average of total OECD member countries was 1,687 hours [5]. Previous studies have shown that long working hours were associated with cerebro-cardiovascular disease [6], hypertension [7], diabetes mellitus (DM) [8], and obesity [9]. Furthermore, a recent cross-sectional study conducted in Korea reported that long working hours were associated with hypothyroidism [10].

According to the Korea National Health and Nutrition Examination Survey (KNHANES VI) from 2013 to 2015, the prevalence of hypothyroidism was 3.8% [11]. Hypothyroidism is a globally common disease [12], which can contribute to cardiovascular disease, cognitive impairment, and chronic kidney disease [13,14]. As the importance of preventing hypothyroidism increases, epidemiological studies of hypothyroidism have reported occupational risk factors, such as shift work [15,16]. Moreover, a recent study using KNHANES VI data demonstrated that hypothyroidism was more prevalent in individuals who worked long hours [10]. However, because of the cross-sectional nature of that study, there was a limitation in elucidating the temporal relationship.

Therefore, we aimed to conduct a longitudinal study to evaluate the causal relationship between long working hours and hypothyroidism by using data from the Kangbuk Samsung Health Study.

## MATERIALS AND METHODS

### Study population

The Kangbuk Samsung Health Study is a cohort study of Koreans aged at least 18 years who underwent annual or biennial comprehensive health screening examinations at the Kangbuk Samsung Hospital Total Healthcare Center in Seoul and Suwon, Korea [17]. Most of the examinees were employees of various companies and local governmental organizations and their spouses. In Korea, the Occupational Safety and Health Act requires annual or biennial health medical examinations to be provided to all employees for free. Other participants underwent health medical check-ups voluntarily at the healthcare center.

**Figure 1:**
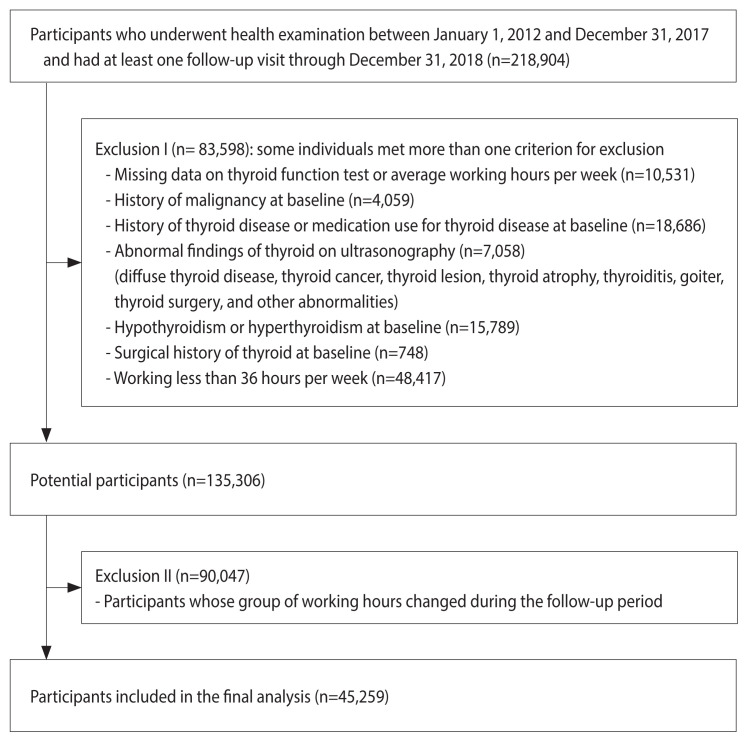
Flow chart of study population with exclusion criteria.

Our study included a total of 218,904 participants who underwent health examinations from January 1, 2012 to December 31 2017, and had experienced at least one other screening exam before December 31, 2018. First, we excluded 83,598 participants who had any of the following conditions at baseline (Figure 1): missing data on thyroid function tests or working hours, history of malignancy, history of thyroid disease or medication use for thyroid disease, abnormal thyroid findings on ultrasonography, hypothyroidism or hyperthyroidism at baseline, surgical history of the thyroid, and working less than 36 hours per week. Second, among the potential participants (135,306 workers), we further excluded 90,047 participants whose category of working hours changed during the follow-up period because of fluctuations in their working hours per week. Finally, 45,259 participants were eligible for the study at baseline.

### Measurements

All examinations were conducted at the Kangbuk Samsung Hospital Total Healthcare Screening Center in Seoul and Suwon. At each visit, information was collected on demographic characteristics, working characteristics, smoking status, alcohol consumption, medical history, and medication use using standardized, self-administered questionnaires [17]. Smoking status was categorized as non-current and current smokers. For alcohol consumption, heavy drinking was categorized as ≥30 g/day for men and ≥20 g/day for women. Cardiovascular disease was defined as a self-reported history of cardiovascular disease, or current use of medications for cardiovascular disease. On the day of the health examination, a trained nurse checked the questionnaire for blanks, and during the last stage of the health examination, a trained doctor double-checked the questionnaire for errors or blanks while conducting a face-to-face interview with the examinee.

Working hours were assessed using the following question: “How many hours did you work in a week on average in your job for the past year, including overtime?” According to the Labor Standards Act of Korea, the working hours of adults should not exceed 40 hours per week except for break time (although 12 additional working hours per week are permitted with employees’ consent). This does not apply to workplaces with fewer than 5 employees, and workers in exceptional industries such as transportation services and health care services are permitted to work more than 52 hours per week. Meanwhile, if a person works more than 60 hours per week for 12 weeks before the onset of cerebro-cardiovascular disease, it is recognized as an occupational disease by the Public Notice of the Ministry of Employment and Labor in Korea. In accordance with the 2021 Labor Statistics of the Korea Labor Institute, a public institution under the Prime Minister, individuals who work less than 36 hours per week are defined as part-time workers. We excluded part-time workers to minimize deviations in evaluating the health effects of long working hours. Based on the above-mentioned standards, the average weekly working hours over the past year were categorized as 36–40 hours, 41–52 hours, 53–60 hours, and >60 hours per week. The shift work schedule was assessed using the following question: “In the past year, during which time of the day did you work the most?” Daytime work was defined as work performed mostly during the day (between 6 a.m. and 6 p.m.), and shift work was defined as work performed during other hours.

Blood pressure, weight, and height were measured by trained nurses [17]. Obesity was defined as body mass index (BMI) ≥25 kg/m^2^. Hypertension was defined as a systolic blood pressure ≥140 mmHg, a diastolic blood pressure ≥90 mmHg, a self-reported history of hypertension, or current use of anti-hypertensive medications. Fasting blood measurements included glucose, thyroid-stimulating hormone (TSH), and free thyroxine (fT4). DM was defined as a fasting serum glucose level of ≥126 mg/dL, a self-reported history of DM, or current use of anti-diabetic medications.

Thyroid function tests were performed routinely since the first visit, regardless of job position or occupation. Serum TSH and fT4 levels were measured by radioimmunoassay using a commercial kit (RIA-gnost^®^ hTSH, fT4, Schering-Cis Bio International, Gif-sur-Yvette, France), with lower detection limits of 0.025 μIU/mL and 0.06 ng/dL, respectively. The normal range was 0.25–5.0 μIU/mL for TSH and 0.93–1.7 ng/dL for fT4. The intra-assay and inter-assay coefficients of variation for quality control specimens were 1.2–5.7% and 2.4–5.4%, respectively, for TSH, and 2.3–4.4% and 2.1–5.7%, respectively, for fT4 [18]. Hypothyroidism was defined as a serum TSH level above the upper reference limit with a normal serum fT4 level (subclinical hypothyroidism) or a serum fT4 level below the lower reference limit (overt hypothyroidism) [10].

### Statistical analysis

The baseline characteristics of study participants were presented according to the 4 groups of weekly working hours. Descriptive statistics were used to summarize the characteristics of participants categorized by the groups of working hours. The primary endpoint was the development of incident hypothyroidism (subclinical or overt hypothyroidism). Participants were followed-up from the baseline to the endpoint visit or to the last available visit until December 31, 2018, whichever came first. Incidence density was calculated as the number of incident cases divided by person-years of follow-up.

Hazard ratios (HRs) and 95% confidence intervals (CIs) for incident hypothyroidism were estimated using Cox proportional hazards regression analyses. Initially, only age and sex were adjusted in the crude model. Model 1 was adjusted for age, sex, alcohol intake, and smoking status. To adjust for the potential confounders, model 2 was further adjusted for DM, hypertension, cardiovascular disease, and BMI. Lastly, to adjust for other occupational risk factors, model 3 was further adjusted for shift work. The proportional-hazards assumption was assessed by examining graphs of estimated log (-log) survival and by using the ‘*estat phtest*’ command based on Schoenfeld residuals; no violation of the assumption was found. To demonstrate the linear trend of incidence, the group number (corresponding to the category of weekly working hours) was used as a continuous variable and examined in each model.

To further explore a dose-response relationship between long working hours and the risk of hypothyroidism, we conducted 2 dose-response analyses. First, HRs were estimated with 95% CIs associated with a 1-hour increase in weekly working hours used as a continuous variable in the regression models. Second, restricted cubic splines with knots were performed at the 5th, 27.5th, 50th, 72.5th, and 95th percentiles of the baseline distribution of weekly working hours. To explore whether the associations between long working hours and hypothyroidism differed, subgroup analyses were performed by age (<36 vs. ≥36 years, stratified by median age), sex (women vs. men), BMI (<25 vs. ≥25 kg/m^2^), and shift work schedule (daytime work vs. shift work). Interactions between the groups of working hours and subgroup characteristics were tested using likelihood ratio tests, which compared models with and without

#### Interaction terms

In a sensitivity analysis to test the robustness of our primary outcomes, we included 90,047 participants whose category of working hours changed during follow-up. Among 135,306 workers, individuals whose category of working hours changed were censored at the time of change. Sequentially, Cox proportional hazards regression analyses and restricted cubic splines with knots were performed in the same way as in the main analysis. Statistical analyses were performed using Stata version 17.0 (StataCorp., College Station, TX, USA). All reported p-values were 2-tailed, and p-values <0.05 were considered statistically significant.

### Ethics statement

This study was approved by the Institutional Review Board of Kangbuk Samsung Hospital, which waived the requirement for informed consent because we accessed only de-identified data routinely collected as part of health screening examinations (IRB No. KBSMC2022-03-041).

## RESULTS

In Table 1, the mean±standard deviation age, serum TSH, and fT4 levels at baseline were 37.6±8.1 years, 2.01±0.97 μIU/mL, and 1.30±0.16 ng/dL, respectively. Weekly working hours were positively associated with male sex, current smoking status, obesity, BMI, systolic blood pressure, diastolic blood pressure, glucose, and fT4 at baseline, and negatively associated with age, hypertension, DM, cardiovascular disease, TSH, and daytime work at baseline.

**Figure 2:**
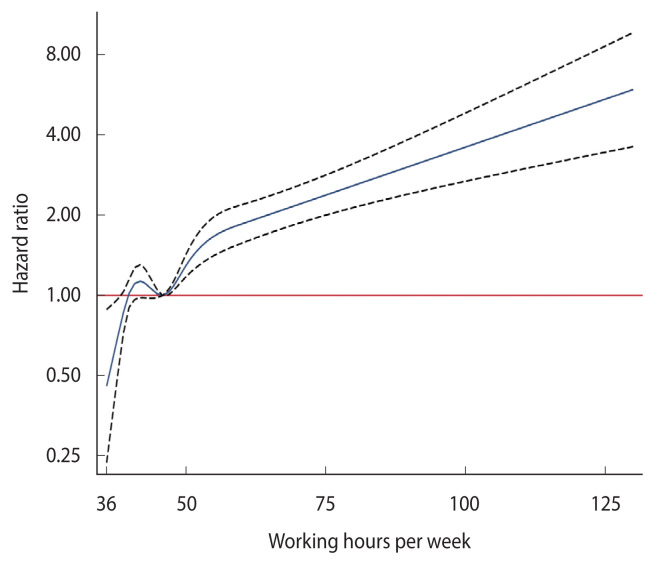
Multivariable-adjusted hazard ratios for hypothyroidism in a restricted cubic spline analysis.

The association between weekly working hours and the risk of incident hypothyroidism is shown in Table 2. In a total of 45,259 participants, there were 2,914 incident cases of hypothyroidism (incidence density, 2.11/10^2^ person-years) over 138,261.7 person-years of follow-up (median follow-up, 2.6 years; interquartile range, 1.9–4.2). All models showed a significantly higher risk of hypothyroidism in all groups that worked more than the reference group (working 36–40 hours per week). In model 3, even after introducing all potential confounders considered in the study, the multivariable-adjusted HRs of incident hypothyroidism for working 41–52 hours, 52–60 hours, and >60 hours compared with working 36–40 hours were 1.13 (95% CI, 1.03 to 1.24), 2.53 (95% CI, 2.17 to 2.95), and 2.57 (95% CI, 2.09 to 3.15), respectively. When weekly working hours were introduced as a continuous variable in regression models, the HR associated with a 1-hour increase in model 3 was 1.02 (95% CI, 1.02 to 1.03). After adjustment in all models, the association remained significant. Increased working hours had a dose-response relationship to the risk of incident hypothyroidism, with a significant trend. Moreover, in the multivariable-adjusted spline regression model, there was a significant dose-response relationship between weekly working hours and incident hypothyroidism at almost the entire range of working hours (Figure 2).

In subgroup analyses (Table 3), a significant association between working more than 52 hours per week and incident hypothyroidism compared with working 36–40 hours per week was consistently shown among participants in all clinically relevant subgroups. However, the association was significantly stronger among participants ≥36 years of age, men, and daytime workers (p for interaction <0.05).

In the sensitivity analysis (Supplementary Material 1), when Cox proportional hazards regression analyses were performed for 135,306 participants, including workers whose category of working hours changed during follow-up, a significant association between working more than 52 hours per week at baseline and incident hypothyroidism compared with working 36–40 hours per week was observed in all models. Furthermore, a significant association between a 1-hour increase in weekly working hours and incident hypothyroidism was shown. In the multivariable-adjusted spline regression model (Supplementary Material 2), a significant and approximately linear dose-response relationship was found between weekly working hours at baseline with incident hypothyroidism across the full range of working hours, except for 40–50 hours.

## DISCUSSION

In the present study wherein the participants had euthyroidism with no thyroid disease at baseline, the results demonstrated that working long hours was associated with the development of hypothyroidism in a dose-response manner. Specifically, our large-scale cohort study showed that regularly working more than 52 hours per week was significantly associated with more than a 2-fold higher risk of incident hypothyroidism compared with regularly working 36–40 hours per week. In addition, the association remained significant even after stratification into predetermined subgroups. When we included workers whose weekly working hours fluctuated, the association was slightly attenuated but still significant. Unlike the previous study by Lee et al. [10], our results could evaluate the temporal effect of long-term exposure to consistent overwork.

To our knowledge, the underlying mechanisms by which long working hours affect the development of hypothyroidism are unknown. Several epidemiological studies investigating the effects of long working hours on health have suggested that the chronic stress response caused by long working hours may play an important role in the biological mechanism [6,19,20]. It is known that long working hours can cause work-related stress such as job strain [3,21]. In turn, chronic psychosocial stress activates the sympathetic nervous system by the hypothalamic-pituitary-adrenal (HPA) axis, stimulates the release of stress hormones such as cortisol, and triggers chronic low-grade inflammation by proinflammatory cytokines [22,23]. As the HPA axis continues to be activated because of chronic stress, HPA axis dysfunction may occur, inducing hypothyroidism by an imbalance of thyroid hormones [24]. In rodent models, repetitive stress triggers the release of cortisol, which suppresses the hypothalamic-pituitary-thyroid (HPT) axis and decreases the peripheral TSH level and thyroid hormone levels [25,26]. A recent epidemiological study by Walter et al. [27] reported a correlation between cortisol and subclinical hypothyroidism. In addition, stress can cause Hashimoto’s thyroiditis by affecting the immune system through the neuroendocrine system (sympathoadrenal and HPA axis) [28]. Posttraumatic stress disorder was found to show a particularly high risk of hypothyroidism, which is explained by inflammatory responses, dysregulation of the HPT axis, and accelerated immune cell aging [29].

As well as biological mechanisms, negative health behaviors, such as obesity, prolonged sedentary time, and physical inactivity, have been suggested as additional mechanisms for the negative health effect of long working hours [3,6]. First, because long working hours are related to obesity and metabolic syndrome [3,9], these conditions may lead to hypothyroidism [30,31]. Notwithstanding such relevance, our results showed a significant association after adjustment for metabolic abnormalities (model 2 in Table 2) and stratification for obesity (Table 3), implying that other mechanisms are still involved. Second, Thorp et al. [32] reported an association between working hours and sedentary time. Prolonged sedentary time at work may also be linked to physical inactivity [6]. In terms of causing changes in circulating thyroid hormone levels according to physical activity and improving thyroid hormone levels in hypothyroidism patients [33,34], it can be hypothesized that hypothyroidism can be caused by physical inactivity [10]. However, since the effect of physical activity on the thyroid is still a matter of debate [35], further studies are required to explain the underlying mechanism.

Considering the significant HRs for covariates in Table 2, previous studies have demonstrated an association between shift work and subclinical hypothyroidism [15,16], but other studies have reported discordant findings [36]. Consistent with several previous studies, our findings showed that shift workers had a higher risk of hypothyroidism than daytime workers (HR, 1.25; 95% CI, 1.10 to 1.42). The main mechanism underlying the relationship between shift work and increased TSH levels appears to be the effect of circadian rhythm disruption [16]. In terms of cigarette smoking, the present study showed that current smoking might have been a protective factor against hypothyroidism (HR, 0.81, 95% CI, 0.73 to 0.90). In keeping with these results, previous studies illustrated that the prevalence of hypothyroidism was significantly lower in current smokers than in non-smokers, indicating that smoking is negatively associated with hypothyroidism [12]. Furthermore, DM was also shown in our results as a protective factor against hypothyroidism (HR, 0.78; 95% CI, 0.62 to 0.98). Regarding treatment for DM, metformin has the effect of lowering TSH levels in DM patients with hypothyroidism or TSH levels in upper normal limits, or who are euthyroid and thyroid peroxidase antibody-positive [37,38]. However, in our study, DM patients who were treated with metformin were not separated from DM patients in general, and there were no data on thyroid peroxidase antibody status. Therefore, further studies are needed to concretely identify the relationship between DM and hypothyroidism.

In subgroup analyses, the association between long working hours and incident hypothyroidism was stronger in older participants and men. It is well-known that old age is a risk factor for hypothyroidism, as serum TSH levels gradually increase with age. The most common reason for hypothyroidism among otherwise healthy elderly individuals is autoimmune thyroiditis [14]. Moreover, with advancing age, hypothalamic dysfunction leads to a realignment of the HPA axis, followed by a decrease in TSH circadian modulation [39]. As long working hours increase stress, the progression of autoimmune thyroiditis and HPA axis dysfunction can be accelerated. Accordingly, it can be inferred that older individuals were more vulnerable to the risk of hypothyroidism with long working hours. However, although shift workers showed a higher risk of hypothyroidism, the risk of long working hours was attenuated among shift workers compared to daytime workers. This may be because shift workers who worked relatively short hours had a high risk of hypothyroidism, and shift workers who worked long hours could have been subject to the healthy worker effect. Therefore, further studies need to be conducted among shift workers.

It is generally known that women are at higher risk of hypothyroidism [14]. This was supported by the present study results (HR, 1.73; 95% CI, 1.57 to 1.91). However, although our study found that long working hours significantly increased the risk of incident hypothyroidism in both men and women, the risk was 3.06-fold higher in men who worked more than 60 hours than in those who worked 36–40 hours, and their risk was significantly higher than that of women (p for interaction=0.009). An explanation for the finding that the risk of long working hours for hypothyroidism was lower for women than for men may be that the reference group of women for working hours was at an elevated risk of hypothyroidism compared to the reference group of men. Additionally, a study by Loosemore & Waters [40] revealed that male workers in the construction industry experienced more stress than women in relation to risk-taking, disciplinary matters, implications of mistakes, redundancy, and career progression. When employees are at high risk of losing their job or threatened with disciplinary action, it is mandatory rather than voluntary for such workers to work long hours [41]. Another study by Rivera-Torres et al. [42] reported that male workers were strongly stressed by quantitative demands, while female workers perceived pressure from qualitative demands (intellectually or emotionally demanding jobs). In this regard, men may be more vulnerable to work-related stress caused by long working hours than women, which might lead to a higher effect of long working hours on hypothyroidism.

Several limitations of the present study need to be considered. First, weekly working hours and several covariates were collected by self-administered questionnaires. The possibility of measurement errors occurring in these variables could not be excluded. However, since there was no disadvantage or benefit to the examinees based on the measured values of these variables, there was no reason to overreport or underreport with a specific intention. Thus, our findings would not be affected by any significant differential misclassification. Second, to clearly identify the health effects of long working hours, we excluded those with a fluctuating category of working hours, which might have caused selection bias. However, a significant association between long working hours and the development of hypothyroidism was observed even when a sensitivity analysis was performed using 135,306 potential participants, including workers whose category of working hours changed during follow-up. Hence, the bias would not have a significant effect on our study. Third, since International Classification of Diseases, 10th revision codes could not be used in our study, the incidence of hypothyroidism was determined by using thyroid function tests. There was a questionnaire about the history of any thyroid disease (not specifying hypothyroidism) at baseline. Therefore, we could exclude all thyroid diseases, including hypothyroidism, at baseline. Since people without any thyroid disease were included when selecting the baseline population, it was assumed that hypothyroidism was first diagnosed through the thyroid function test. Fourth, the effect of interval censoring could not be ignored. However, in our study, the average health check-up interval was the same between workers who worked over 52 hours per week and workers who worked less than 52 hours (1.8 vs. 1.8 years). Therefore, differences in the likelihood of detecting hypothyroidism due to long working hours are unlikely to have affected our study results. Fifth, since thyroid function tests were not remeasured 6 months to 1 year after the first measurement, temporary thyroid dysfunction and secondary dysfunction could not be completely ruled out. Moreover, our primary outcomes included subclinical hypothyroidism, as well as overt hypothyroidism, and the age-specific references of thyroid function tests for hypothyroidism were not used. Thus, the incidence density of hypothyroidism in our study would be higher than that in the general population. Sixth, thyroid peroxidase antibody, iodine diet, type of worker (e.g., blue-collar vs. white-collar), occupational stress, and other lifestyle variables were not evaluated in this study. Studying these factors in further research may contribute to elucidating the mechanisms underlying our results. According to the Occupational Safety and Health Act of Korea, a business owner should provide a general health check-up to office workers (white-collar) biennially and to non-office workers annually. When subgroup analysis was performed according to the average health check-up interval (Supplementary Material 3), the risk of hypothyroidism was higher when annual health check-ups were received. This could have been due to exposure to various hazardous substances in blue-collar occupations [43,44]. However, if workers do not receive a health check-up, it can be carried over to the next year, and it is possible to receive an examination at another hospital. Therefore, we could not classify workers as blue-collar or white-collar based on the interval between check-ups. Meanwhile, the extent to which a potential unmeasured confounder negated the observed association between long working hours and hypothyroidism can be assessed by calculating the E-value [45]. In model 3, the measured E-value for working more than 60 hours per week was 4.58 (95% CI, 3.60 to 5.75), suggesting that the observed HR of 2.57 could be explained away by an unmeasured confounder that was associated with both our exposure and outcome by an HR of 4.58-fold each, above and beyond the measured confounders, but weaker confounding could not do so; furthermore, the 95% CI could be shifted to include the null by an unmeasured confounder that was associated with both our exposure and outcome by an HR of 3.60-fold each, above and beyond the measured confounders, but weaker confounding could not do so [45]. The E-value in the present study indicates the robustness of our main findings to unmeasured confounding. Lastly, the participants were young and middle-aged Koreans in relatively good health. Accordingly, our results may not be generalizable to other populations differing in terms of age, race, and ethnicity.

Notwithstanding the abovementioned limitations, this study has several notable strengths. To the best of our knowledge, this is the first longitudinal study with a large-scale sample size, well-designed cohort, and standardized data to demonstrate the temporal association between long working hours and the development of hypothyroidism. Moreover, by examining a fixed exposure through repeated measurements of working hours, the study examined the effect of long working hours more clearly than before. In this study, only subjects with no change in the category working hours were included to compensate for the inaccuracy of self-questionnaires and to accurately estimate risk by selecting only individuals with constant exposure for reliable survival analysis. However, since the healthy worker effect could have been applicable to those whose working hours did not change, the actual effect might have been underestimated. Therefore, the actual risk might be higher than the results of this study. Lastly, the study was based on a relatively young and middle-aged healthy population, implying less susceptibility to survivor bias from comorbidities.

In conclusion, our large-scale cohort study demonstrated the association between long working hours and the incidence of hypothyroidism, with a dose-response relationship. As the impacts of long working hours continue to be revealed, more attention should be paid to the health of overworked workers.

## Figures and Tables

**Table 1.** Baseline characteristics of study participants by weekly working hours without changing group of weekly working hours

Characteristics	Overall	Weekly working hours				p-value for trend
		36-40	41-52	53-60	>60	
Total (n)	45,259	15,139	26,327	2,545	1,248	
Age (yr)	37.6±8.1	40.6±8.9	36.0±7.1	37.4±7.6	37.1±8.5	<0.001
Men	69.8	52.4	78.0	82.2	82.6	<0.001
Current smoker	22.5	19.0	22.9	32.3	35.5	<0.001
Alcohol heavy intake^1^	17.0	17.8	15.9	21.0	22.0	0.419
Hypertension	11.3	13.0	10.2	13.4	10.6	<0.001
Diabetes mellitus	3.6	5.0	2.8	3.4	3.8	<0.001
Cardiovascular disease	1.0	1.5	0.8	0.9	1.4	0.003
Obesity^2^	31.7	27.7	32.9	38.6	38.5	<0.001
BMI (kg/m^2^)	23.7±3.3	23.2±3.3	23.8±3.3	24.3±3.4	24.4±3.5	<0.001
Systolic BP (mmHg)	109.5±12.7	107.7±13.4	110.2±12.3	111.4±12.5	111.3±12.3	<0.001
Diastolic BP (mmHg)	70.8±10.1	70.2±10.6	71.1±9.8	71.9±10.1	71.2±9.6	<0.001
Glucose (mg/dL)	93 (88-99)	93 (88-100)	93 (88-99)	94 (88-99)	94 (89-100)	0.001
TSH (μIU/mL)	2.01±0.97	2.01±0.97	2.01±0.97	1.97±0.98	1.98±0.99	0.045
free T4 (ng/dL)	1.30±0.16	1.27±0.15	1.32±0.15	1.31±0.16	1.30±0.16	<0.001
Daytime work^3^	89.9	91.8	89.5	88.7	76.5	<0.001

Values are presented as mean±standard deviation, percentage, or median (interquartile range). BMI, body mass index; BP, blood pressure; TSH, thyroid-stimulating hormone.

1 Women: ≥20 g/day, men: ≥30 g/day.

2 BMI ≥25kg/m^2^.

3 Participants who answered “I work mostly during the day (between 6 a.m. and 6 p.m.)”.

**Table 2.** Development of hypothyroidism according to weekly working hours without changing group of working hours including hazard ratios of covariates

Variables		PY	Incident cases	Incidence density (per 10^2^ PY) (95% CI)	Age- and sex-adjusted	Multivariable-adjusted^1^		
						Model 1	Model 2	Model 3
Weekly working hours								
	36-40	46,576.5	1,084	2.32 (2.19, 2.47)	1.00 (reference)	1.00 (reference)	1.00 (reference)	1.00 (reference)
	41-52	82,653.6	1,484	1.80 (1.71, 1.89)	1.10 (1.01, 1.20)	1.12 (1.02, 1.22)	1.12 (1.02, 1.23)	1.13 (1.03, 1.24)
	53-60	5,992.5	228	3.80 (3.34, 4.33)	2.44 (2.11, 2.83)	2.50 (2.15, 2.91)	2.51 (2.15, 2.92)	2.53 (2.17, 2.95)
	>60	3,039.1	118	3.88 (3.24, 4.65)	2.42 (1.99, 2.93)	2.57 (2.10, 3.14)	2.56 (2.09, 3.12)	2.57 (2.09, 3.15)
	Per 1 hour				1.02 (1.02, 1.03)	1.02 (1.02, 1.03)	1.02 (1.02, 1.03)	1.02 (1.02, 1.03)
	p for trend				<0.001	<0.001	<0.001	<0.001
Age					1.04 (1.03, 1.04)	1.04 (1.03, 1.05)	1.04 (1.04, 1.05)	1.04 (1.04, 1.05)
Sex								
	Men				1.00 (reference)	1.00 (reference)	1.00 (reference)	1.00 (reference)
	Women				1.81 (1.67, 1.97)	1.71 (1.56, 1.88)	1.75 (1.58, 1.93)	1.73 (1.57, 1.91)
Alcohol intake								
	Not heavy drinking					1.00 (reference)	1.00 (reference)	1.00 (reference)
	Heavy drinking					1.05 (0.95, 1.17)	1.05 (0.95, 1.17)	1.05 (0.94, 1.17)
Smoking status								
	Not current smoker					1.00 (reference)	1.00 (reference)	1.00 (reference)
	Current smoker					0.82 (0.74, 0.91)	0.82 (0.74, 0.91)	0.81 (0.73, 0.90)
DM							0.79 (0.63, 0.98)	0.78 (0.62, 0.98)
Hypertension							0.98 (0.86, 1.12)	0.97 (0.85, 1.10)
Cardiovascular disease							1.05 (0.73, 1.51)	1.01 (0.70, 1.46)
BMI							1.01 (1.00, 1.02)	1.01 (0.99, 1.02)
Shift work schedule								
	Daytime work							1.00 (reference)
	Shift work							1.25 (1.10, 1.42)

Values are presented as hazard ratio (95% CI). PY, person-years; CI, confidence interval; DM, diabetes mellitus; BMI, body mass index.

1 Estimated from Cox proportional hazard models; Model 1 was adjusted for age, sex, alcohol intake, and smoking status; Model 2: model 1 plus adjustment for hypertension, DM, cardiovascular disease, and BMI; Model 3: model 2 plus adjustment for shift work.

**Table 3.** Hazard ratios (95% CI) for hypothyroidism by weekly working hours in clinically relevant subgroups without changing group of working hours^1^

Subgroup		Weekly working hours				p for trend	p for interaction
		36-40	41-52	53-60	>60		
Age (yr)							0.009
	<36 (n=21,659)	1.00 (reference)	0.98 (0.83, 1.14)	2.21 (1.69, 2.89)	2.32 (1.64, 3.28)	<0.001	
	≥36 (n=23,600)	1.00 (reference)	1.17 (1.04, 1.31)	2.65 (2.19, 3.19)	2.73 (2.12, 3.52)	<0.001	
Sex							0.009
	Women (n=13,667)	1.00 (reference)	1.06 (0.92, 1.22)	1.77 (1.27, 2.48)	1.65 (1.02, 2.69)	0.004	
	Men (n=31,592)	1.00 (reference)	1.22 (1.08, 1.38)	2.93 (2.44, 3.52)	3.06 (2.42, 3.87)	<0.001	
Body mass index (kg/m^2^)							0.259
	<25 (n=30,903)	1.00 (reference)	1.10 (0.99, 1.23)	2.31 (1.90, 2.81)	2.40 (1.84, 3.13)	<0.001	
	≥25 (n=14,323)	1.00 (reference)	1.21 (1.01, 1.44)	2.97 (2.29, 3.85)	2.93 (2.11, 4.07)	<0.001	
Shift work schedule							0.025
	Daytime work (n=40,674)	1.00 (reference)	1.13 (1.02, 1.24)	2.57 (2.19, 3.02)	2.93 (2.35, 3.66)	<0.001	
	Shift work (n=4,238)	1.00 (reference)	1.06 (0.79, 1.41)	2.09 (1.23, 3.56)	1.36 (0.79, 2.35)	0.070	

1 Estimated from Cox proportional hazard models adjusted for age, sex, alcohol intake, smoking status, hypertension, diabetes mellitus, cardiovascular disease, body mass index, and shift work.
