# Supplementary material for: Long working hours and the risk of hypothyroidism in healthy Korean workers: a cohort study
Source: Epidemiol Health. 2022 Nov 8;44:e2022104. doi: 10.4178/epih.e2022104 (PMC10106547; doi:10.4178/epih.e2022104)
Supplement: Supplementary file 1 [file epih-44-e2022104-Supplementary-1.docx]

| **Supplementary Material 1. Development of hypothyroidism according to weekly working hours among whole group including participants censored due to changes of working hours** | | | | | | | |
| --- | --- | --- | --- | --- | --- | --- | --- |
| Weekly working hours | Person-years (PY) | Incident cases | Incidence density  (per 10^2^ PY) (95% CI) | Age- and sex-adjusted HR (95% CI) | Multivariable-adjusted HR (95% CI)^a^ | | |
|  |  |  |  |  | Model 1^*^ | Model 2^**^ | Model 3^***^ |
| Hypothyroidism | |  |  |  |  |  |  |
| 36-40 | 77,101.4 | 1,425 | 1.85 (1.75-1.95) | 1.00 (reference) | 1.00 (reference) | 1.00 (reference) | 1.00 (reference) |
| 41-52 | 188,554.0 | 2,510 | 1.33 (1.28-1.38) | 0.98 (0.91-1.05) | 0.99 (0.92-1.06) | 0.99 (0.92-1.07) | 1.00 (0.93-1.07) |
| 53-60 | 56,464.7 | 738 | 1.31 (1.22-1.40) | 1.12 (1.02-1.23) | 1.13 (1.03-1.25) | 1.13 (1.03-1.25) | 1.15 (1.04-1.26) |
| >60 | 23,301.6 | 327 | 1.40 (1.26-1.56) | 1.22 (1.08-1.38) | 1.27 (1.12-1.44) | 1.27 (1.12-1.44) | 1.26 (1.11-1.44) |
| per 1 hour |  |  |  | 1.01 (1.003-1.01) | 1.01 (1.004-1.01) | 1.01 (1.004-1.01) | 1.01 (1.004-1.01) |
| *P* for trend |  |  |  | <0.001 | <0.001 | <0.001 | <0.001 |
| ^a^ Estimated from Cox proportional hazard models. | | | | | | | |
| ^*^ Model 1 was adjusted for age, sex, alcohol intake, and smoking status. | | | | | | | |
| ^**^ Model 2: model 1 plus adjustment for hypertension, DM, cardiovascular disease, and BMI. | | | | | | | |
| ^***^ Model 3: model 2 plus adjustment for shift work. | | | | | | | |
| HR, hazard ratio; CI, confidence interval; DM, diabetes mellitus; BMI, body mass index | | | | | | | |
