# Supplementary material for: Long working hours and the risk of hypothyroidism in healthy Korean workers: a cohort study
Source: Epidemiol Health. 2022 Nov 8;44:e2022104. doi: 10.4178/epih.e2022104 (PMC10106547; doi:10.4178/epih.e2022104)
Supplement: Supplementary file 2 [file epih-44-e2022104-Supplementary-2.docx]

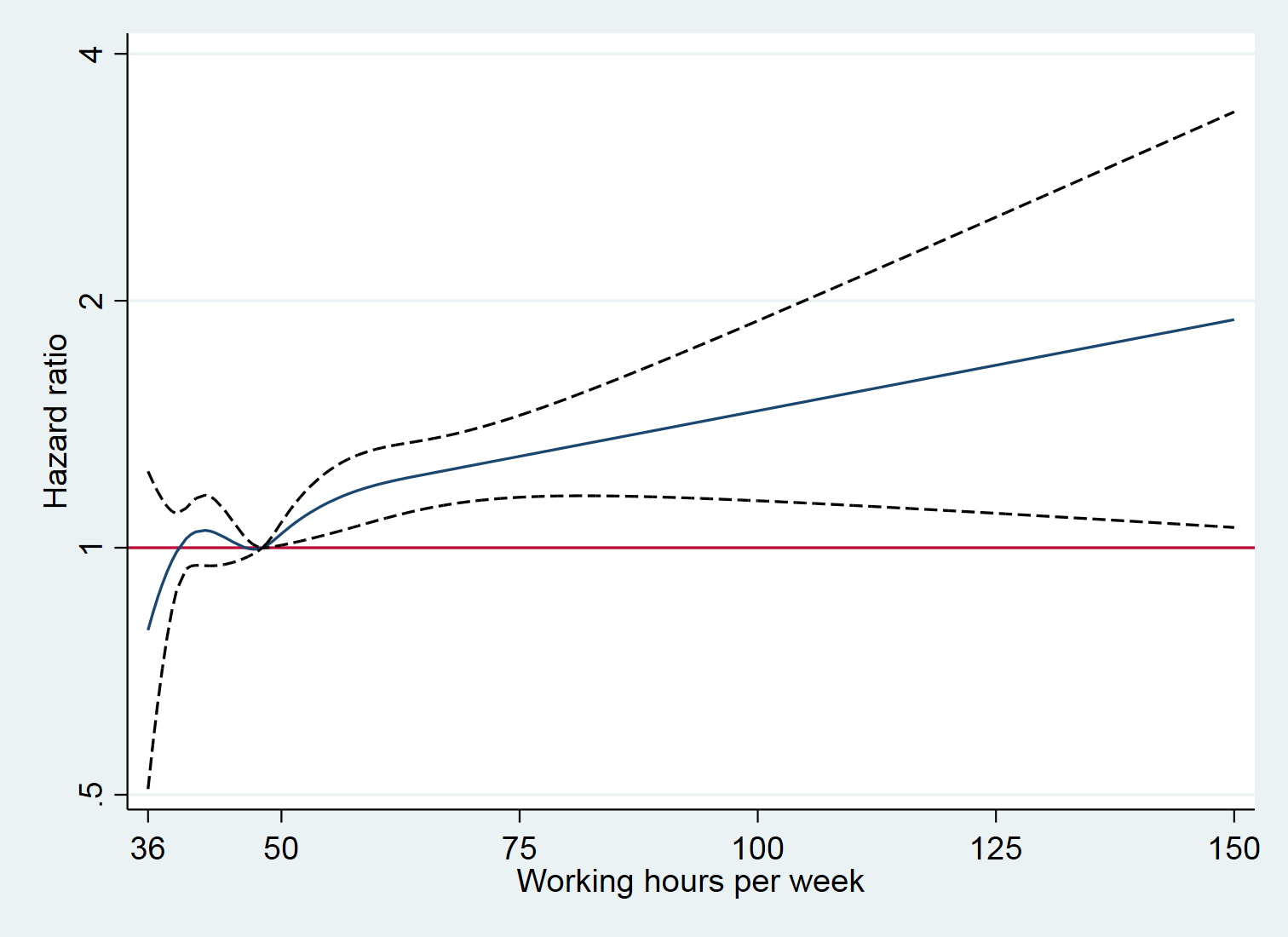


**Supplementary Material 2. Multivariable-adjusted hazard ratio for hypothyroidism with the restricted cubic spline among the censored due to changes of working hours**
