# Supplementary material for: Long working hours and the risk of hypothyroidism in healthy Korean workers: a cohort study
Source: Epidemiol Health. 2022 Nov 8;44:e2022104. doi: 10.4178/epih.e2022104 (PMC10106547; doi:10.4178/epih.e2022104)
Supplement: Supplementary file 3 [file epih-44-e2022104-Supplementary-3.docx]

| **Supplementary Material 3. Hazard ratios^a^ (95% CI) for hypothyroidism by weekly working hours in subgroups by the average of health check-up intervals without changing group of working hours** | | | | | | |
| --- | --- | --- | --- | --- | --- | --- |
| Subgroup | Weekly working hours | | | | *P* for trend | *P* for interaction |
|  | 36-40 | 41-52 | 53-60 | >60 |  |  |
| Average of health check-up intervals during follow-up period |  |  |  |  |  | <0.001 |
| ≤1 years (n=10,326) | 1.00 (reference) | 1.16 (0.98-1.36) | 2.86 (2.20-3.71) | 2.85 (2.04-3.97) | <0.001 |  |
| >1 years (n=34,933) | 1.00 (reference) | 1.08 (0.96-1.21) | 2.39 (1.97-2.89) | 2.29 (1.76-2.98) | <0.001 |  |
| ^a^ Estimated from Cox proportional hazard models adjusted for age, sex, alcohol intake, smoking status, hypertension, DM, cardiovascular disease, BMI, and shift work | | | | | | |
| CI, confidence interval; DM, diabetes mellitus; BMI, body mass index | | | | | | |
